# The Role of Beta-Endorphin in Cocaine-Induced Conditioned Place Preference, Its Extinction, and Reinstatement in Male and Female Mice

**DOI:** 10.3389/fnbeh.2021.763336

**Published:** 2021-12-10

**Authors:** Prableen K. Singh, Kabirullah Lutfy

**Affiliations:** Department of Pharmaceutical Sciences, College of Pharmacy, Western University of Health Sciences, Pomona, CA, United States

**Keywords:** cocaine, beta-endorphin, conditioned place preference, extinction, reinstatement, sex

## Abstract

Endogenous opioids have been implicated in cocaine reward. However, the role of each opioid peptide in this regard is unknown. Notably, the role of each peptide in extinction and reinstatement is not fully characterized. Thus, we assessed whether cocaine-induced conditioned place preference (CPP) and its extinction and reinstatement would be altered in the absence of beta-endorphin. We also examined if sex-related differences would exist in these processes. Male and female mice lacking beta-endorphin and their respective controls were tested for baseline place preference on day 1. On day 2, mice were treated with saline/cocaine (15 mg/kg) and confined to the vehicle- or drug-paired chamber for 30 min, respectively. In the afternoon, mice were treated with the alternate treatment and confined to the opposite chamber. Mice were then tested for CPP on day 3. Mice then received additional conditioning on this day as well as on day 4. Mice were then tested for CPP on day 5. Mice then received extinction training on day 9. On day 10, mice were tested for extinction and then reinstatement of CPP following a priming dose of cocaine (7.5 mg/kg). Male and female mice lacking beta-endorphin did not exhibit CPP following single conditioning with cocaine. On the other hand, only male mice lacking beta-endorphin failed to show CPP after repeated conditioning. Nonetheless, reinstatement of CPP was blunted in both male and female mice lacking beta-endorphin compared to controls. The present results suggest that beta-endorphin plays a functional role in cocaine-induced CPP and its reinstatement, and sex-related differences exist in the regulatory action of beta-endorphin on the acquisition but not reinstatement of cocaine CPP.

## Introduction

Cocaine addiction is a serious public health and socioeconomic challenge in the United States and many other countries around the globe. In 2001, it was estimated that roughly 1.2 million people in the United States consumed cocaine for the very first time (O’Brien and Anthony, 2005). In addition, in 2007, over 2 million people over the age of 12 were current cocaine users, and females were found to be more susceptible to the addictive properties of cocaine (Kasperski et al., 2011). The prevalence of cocaine addiction in Europe and Latin America and other countries including the United States is of a major concern (Castells et al., 2016). Yet, there is no medication to effectively treat cocaine addiction (Castells et al., 2016; Pierce et al., 2018).

Opioid peptides (beta-endorphin, enkephalin, and dynorphin) and receptors (mu, delta, and kappa opioid receptors) are expressed in the central nervous system and are known to play a functional role in motivated behaviors, natural reward, and most importantly, drug reward (Shippenberg and Herz, 1986, 1987; Spanagel et al., 1990, 1992; Skoubis et al., 2001, 2005). For example, opioid receptor agonists increase the rewarding and reinforcing actions of psychostimulants, which have been demonstrated using self-administration and conditioned place preference (CPP) paradigms (Le Merrer et al., 2009). In contrast, opioid receptor antagonists have been shown to decrease the rewarding and reinforcing actions of cocaine (Houdi et al., 1989; Ramsey and van Ree, 1991; Suzuki et al., 1992; Gerrits et al., 1995; Kim et al., 1997; Rademacher and Steinpreis, 2002; Hummel et al., 2006), suggesting that endogenous opioid peptides may regulate these actions of cocaine. However, the role of each opioid peptide in the rewarding and reinforcing action of cocaine is not fully characterized.

Several studies have shown that beta-endorphin plays a functional role in the reinforcing actions of cocaine. For example, cocaine has been shown to cause the release of beta-endorphin in the nucleus accumbens (NAc; Olive et al., 2001; Roth-Deri et al., 2003, 2008), a response shown to be involved in cocaine self-administration (Roth-Deri et al., 2003, 2008). Endogenous beta-endorphin may also be involved in the rewarding action of cocaine. For instance, we have previously shown that the rewarding action of cocaine, using a single conditioning paradigm, was reduced in mice lacking beta-endorphin compared to their wild-type controls (Marquez et al., 2008; Nguyen et al., 2012), suggesting beta-endorphin may play a functional role in the rewarding action of acute cocaine. However, whether beta-endorphin plays a functional role in the rewarding action of cocaine after repeated administration of the drug is unclear.

Preclinical studies demonstrated that female rats develop cocaine-induced conditioned place preference (CPP) faster than male rats (for a review, see Becker and Koob, 2016). Female rats also acquire CPP at lower doses of cocaine (5, 10 mg/kg versus 20 mg/kg) than male rats (Russo et al., 2003). Sex-related differences have also been reported following intermittent- and long-term cocaine self-administration in adult rats (Algallal et al., 2020). This sex-related difference is also observed in adolescent (PND34) rats (Zakharova et al., 2009). However, the underlying mechanism of this sex-related difference in cocaine reward remains mostly unknown. Therefore, we also determined if sex-related differences exist in the rewarding action of cocaine and if endogenous beta-endorphin plays a functional role in cocaine reward after its acute or repeated administration.

Relapse represents a serious issue in the treatment of drug addiction. Craving can be easily triggered by any drug-associated cues, external environmental cues, or re-exposure to the drug itself (Bossert et al., 2013). Thus, understanding the underlying mechanisms of craving and relapse may aid in the development of medications to manage addiction. There is some evidence showing beta-endorphin is involved in extinction and drug seeking behaviors (del Cerro and Borrell, 1987; Roth-Deri et al., 2004; Simmons and Self, 2009). Thus, in the present study, we also determined the role of beta-endorphin in extinction and reinstatement. Considering the place conditioning paradigm is used as a measure of reward, extinction, and reinstatement (Bardo and Bevins, 2000; Mueller and Stewart, 2000; Botreau et al., 2006; Carey et al., 2007; Jackson et al., 2013), we used this paradigm to characterize the role of endogenous beta-endorphin in cocaine-induced CPP and its extinction and reinstatement. We also assessed if sex-related differences would exist in the regulatory action of beta-endorphin in these processes.

## Materials and Methods

### Subjects

A total of 14 male and 16 female mice lacking beta-endorphin (Rubinstein et al., 1996) and their wild-type littermates/age-matched controls (*n* = 6–8 mice per genotype of each sex) bred in-house were used for all the experiments. A number of studies showed that there was no compensatory changes in the diurnal corticosterone levels, and other component of the hypothalamic-pituitary-adrenal axis (Rubinstein et al., 1996). Furthermore, the expression of opioid receptors in different brain regions and spinal cord (Mogil et al., 2000) or the total brain levels of mu opioid receptors (Slugg et al., 2000) were unchanged in these mice compared to their wild-type controls. The latter study also showed normal coupling of the mu opioid receptors to the potassium channels (Slugg et al., 2000). Moreover, the distribution and level of opioid peptides enkephalins and dynorphins seemed to be unchanged in mice lacking beta-endorphin compared to their wild-type controls (Rubinstein et al., 1996). The breeding pairs were originally obtained from Jackson Laboratories (Bar Harbor, ME, United States). Pups were weaned at the age of 21 days and genotyped a few days later, as described in our earlier report (Tseng et al., 2013). Mice were housed 2–4 per cage with the same-sex littermate. Subjects were maintained in a temperature-controlled environment (22 ± 1°C) under a 12-h light/dark cycle (6 am – 6 pm) and had free access to food and water in their home cages. All behavioral experiments were conducted during the light cycle between 9 am and 4 pm. All experimental procedures were according to the NIH guidelines for the care and use of animals in research and approved by the Institutional Animal Care and Use Committee at Western University of Health Sciences (Pomona, CA, United States).

### Drugs

Cocaine hydrochloride purchased from Sigma-Aldrich (St. Louis, MO, United States) was dissolved in saline and administered intraperitoneally.

### Experimental Procedures

#### To Determine the Role of Beta-Endorphine and Sex in Cocaine-Induced Conditioned Place Preference and Its Extinction and Reinstatement

The place conditioning paradigm has been used as a behavioral assay in preclinical studies to assess the rewarding actions of cocaine and other addictive drugs (Bardo and Bevins, 2000). This paradigm has also been used as an animal model of extinction and reinstatement because, like any other conditioned response, CPP can be extinguished and reinstated (Sanchez and Sorg, 2001; Itzhak and Martin, 2002; Kelley et al., 2007). We used a three-chambered place conditioning apparatus (ENV-3013, Med Associates Inc., Saint Albans, VT, United States) and an unbiased paradigm to examine the role of sex and beta-endorphin in cocaine-induced CPP and its extinction and reinstatement. The details of the procedure and apparatus have been provided elsewhere (Nguyen et al., 2012; Tseng et al., 2013). The place conditioning protocol consisted of three phases and was conducted over 10 days, as depicted in diagram below: the acquisition phase, extinction phase, and reinstatement phase.


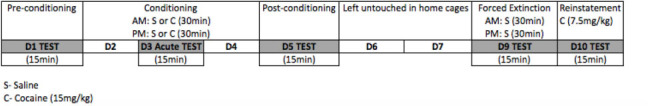


#### The Role of Beta-Endorphin and Sex in the Acquisition of Conditioned Place Preference Following Single Conditioning With Cocaine

We first used single conditioning with cocaine to assess the role of beta-endorphin in the rewarding action of acute cocaine. To this end, mice (6–8 mice of each sex per genotype) were tested for preconditioning (or baseline) place preference on day 1 (D1). On this day, each mouse was placed in the central neutral chamber and allowed to roam the conditioning and neutral central chambers freely for 15 min. The amount of time, that mice spent in the three chambers, was recorded via MED-PC IV (Med Associates, Inc.). On day 2, animals were treated with cocaine (15 mg/kg, i.p.) or saline and confined to the drug-paired chamber (DPCh) or vehicle-paired chamber (VPCh) for 30 min. In the afternoon, mice received the alternate treatment and were confined in the opposite chamber for 30 min. Mice were then tested for place preference on day 3 (D3), as described for day 1.

#### The Role of Beta-Endorphin and Sex in the Acquisition, Extinction and Reinstatement of Conditioned Place Preference Induced by Repeated Conditioning With Cocaine

To determine the role of beta-endorphin in cocaine reward after repeated conditioning, shortly after the test, mice received their respective twice-daily conditioning on this day as well as on day 4. On day 5 (D5), animals were tested again for place preference after repeated conditioning with cocaine. After that, mice were left undisturbed in their home cages on days 6 and 7 and then tested for natural extinction on day 8 (data not shown). To determine the role of beta-endorphin in extinction, animals received forced extinction training on day 9, in which mice were conditioned with saline in both conditioning chambers. On day 10, mice were tested for extinction, as described on day 1, in the morning (between 10 and 11 AM). Animals were considered to express extinction when there was no significant difference between the amount of time that mice spent in the drug-paired (DPCh) versus vehicle-paired (VPCh) chamber. In the afternoon (between 2 and 3 PM), mice were tested for the reinstatement of cocaine CPP immediately following a single cocaine injection (7.5 mg/kg, i.p.). On each test day, mice were placed in the neutral chamber and allowed to freely explore the three chambers for 15 min. The amount of time that mice spent in each chamber was recorded on each test day.

### Data Analysis

The data are presented as means (±SEM) of the amount of time that animals spent in drug-paired (DPCh) versus vehicle-paired chamber (VPCh). A three-way repeated-measure analysis of variance (ANOVA) was performed, followed by the Fisher’s LSD *post hoc* test for multiple comparisons. A *P* ≤ 0.05 was considered statistically significant.

## Results

### Acquisition and Reinstatement of CPP Were Blunted in Male Mice Lacking Beta-Endorphin Compared to Their Wild-Type Controls

**FIGURE 1:**
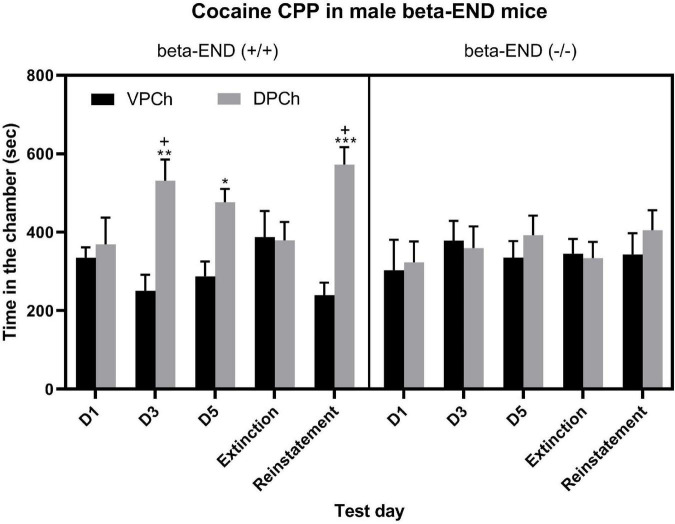
CPP was blunted in male mice lacking beta-endorphin compared to their wild-type controls following single and repeated conditioning with cocaine (15 mg/kg) as well as on the reinstatement test day following a challenge dose of cocaine (7.5 mg/kg, i.p.). Data are mean (±SEM) of the amount of time that animals (*n* = 6–8 mice per genotype) spent in the drug-paired chamber (DPCh) versus vehicle-paired chamber (VPCh) before (day 1; D1) and after single (day 3; D3) and repeated (day 5; D5) conditioning with cocaine as well as on the extinction and reinstatement test days. *P < 0.05; **P < 0.01; ***P < 0.001 vs. its respective VPCh; +*P* < 0.05 vs. mice lacking beta-END on that test day.

The amount of time that male beta-endorphin wild-type (left half of the panel) and knockout (right half of the panel) mice spent in the vehicle-paired (VPCh) and drug-paired (DPCh) chambers on the baseline preference test day (day 1; D1) as well as on test days for CPP after single conditioning on day 3 (D3), day 5 (D5) and on extinction and reinstatement (Figure 1). A three-way repeated-measure ANOVA revealed a significant effect of genotype [*F*_(1,55)_ = 4.22; *P* < 0.05], a significant effect of context [*F*_(1,55)_ = 11.10; *P* < 0.01] and a significant interaction between the two factors [*F*_(1,55)_ = 6.51; *P* < 0.01]. The *post hoc* test showed a significant difference in the amount of time that wild-type mice (beta-END+/+) spent in the DPCh vs. VPCh on the postconditioning (D3 or D5) as well as on the reinstatement test day (*P* < 0.05) but not before conditioning (D1) or the extinction test day (Figure 1, left half of the panel). On the other hand, the beta-endorphin knockout mice did not exhibit any CPP response following either single or repeated conditioning with cocaine (Figure 1, right half of the panel). There was no significant (*P* > 0.05) difference between the amount of time that mice spent in the DPCh vs. VPCh following the challenge dose of cocaine on the reinstatement test day in mice lacking beta-endorphin (beta-END−/−). The *post hoc* analyses of the data showed that there was a significant difference in the amount of time that wild-type mice spent in the DPCh on the postconditioning test days as well as on the reinstatement test day (*P* < 0.05). These results show that the acquisition of cocaine-induced CPP after single and repeated conditionings is reduced in the absence of beta-endorphin. Likewise, the reinstatement of CPP was blunted in the absence of the peptide.

### Reinstatement of Cocaine-Induced CPP and Its Acquisition Following Single but Not Repeated Conditioning With Cocaine Were Reduced in Female Mice Lacking Beta-Endorphin Compared to Their Wild-Type Controls

**FIGURE 2:**
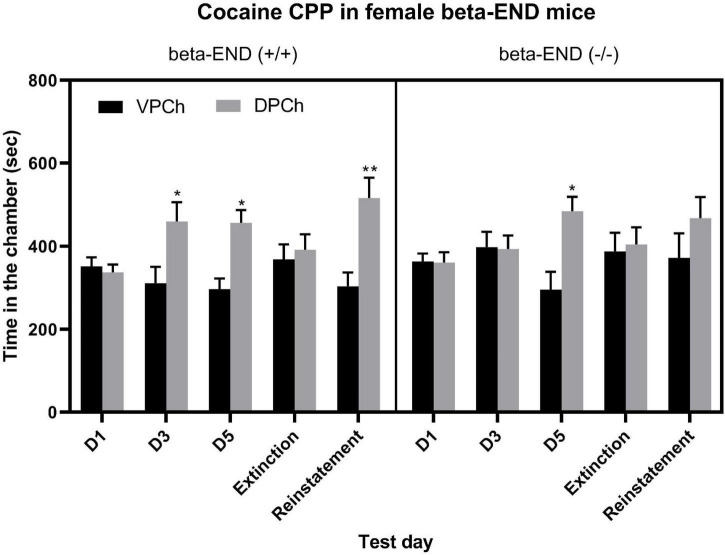
The CPP response was blunted in female mice lacking beta-endorphin compared to their wild-type controls following single but not repeated conditioning with cocaine (15 mg/kg) as well as on the reinstatement test day. Data are mean (±SEM) of the amount of time that mice (*n* = 8 mice per genotype) spent in the drug-paired chamber (DPCh) versus vehicle-paired chamber (VPCh) on day 1 (D1) and after single (day 3; D3) and repeated (day 5, D5) conditioning with cocaine as well as on the extinction and reinstatement test days; *P < 0.05; **P < 0.01 vs. their respective VPCh on that day.

Figure 2 shows the amount of time that female wild-type (left half of the panel) and beta-endorphin knockout (right half of the panel) mice spent in the conditioning chambers before (D1) and after single (D3) and repeated (D5) cocaine conditioning as well as on the extinction and reinstatement test days. A three-way repeated-measure ANOVA revealed a significant effect of genotype [*F*_(1,70)_ = 3.86; *P* = 0.05], a significant effect of context [*F*_(1,70)_ = 12.85; *P* < 0.001], but there was no significant interaction between the two factors [*F*_(1,70)_ = 1.06; *P* > 0.05]. The *post hoc* test showed a significant (*P* < 0.05) increase in the amount of time that female wild-type (beta-END+/+) mice spent in the DPCh vs. VPCh on the postconditioning test days (D3 and D5) as well as on the reinstatement test day (Figure 2, left half of the panel). On the other hand, mice lacking beta-endorphin (beta-END−/−) failed to exhibit any CPP after the single conditioning with cocaine (Figure 2, D3, right half of the panel). Interestingly, these mice showed a significant CPP after repeated conditioning with cocaine. Yet, they failed to show a significant reinstatement (Figure 2, D5, right half panel). These results indicate that female wild-type mice exhibited a significant CPP following single and repeated cocaine conditioning. On the other hand, beta-endorphin knockout mice showed a significant CPP response only after repeated cocaine conditioning. Furthermore, the reinstatement of CPP was reduced in mice lacking beta-endorphin compared to their wild-type controls.

## Discussion

The main findings of the present study are that the rewarding action of acute cocaine was reduced in both male and female mice lacking beta-endorphin. Likewise, the CPP response induced by repeated cocaine conditioning was attenuated in male, but not female mice lacking beta-endorphin. In mice undergone extinction training, cocaine was able to reinstate the CPP response in both male and female wild-type but not knockout mice. Together, these results suggest that beta-endorphin plays a functional role in the rewarding action of cocaine following single and repeated conditioning but there seem to be some sex-related differences in this response. However, beta-endorphin plays an essential role in the processes leading to the reinstatement of cocaine CPP regardless of the sex of mice.

Cocaine has been shown to cause the release of beta-endorphin in the NAc (Olive et al., 2001; Roth-Deri et al., 2003), a response that may be important in the acquisition of cocaine self-administration (Roth-Deri et al., 2004, 2006, 2008) as well as in incubation processes (Dikshtein et al., 2013). Interestingly, we have previously shown that the rewarding action of acute cocaine was reduced in male mice lacking beta-endorphin compared to their wild-type controls (Marquez et al., 2008; Nguyen et al., 2012). In the present study, we wanted to extend these findings to female mice as well as assessed the role of beta-endorphin in CPP induced by repeated cocaine conditioning in both male and female mice. Previous studies have demonstrated that there are sex-related differences in the acquisition of cocaine CPP (Hilderbrand and Lasek, 2014). For example, females have been demonstrated to acquire cocaine CPP with fewer conditioning sessions and at lower doses than males (Russo et al., 2003). On the contrary, reports are showing no sex-related differences in cocaine reward using a wide range of doses (3–25 mg/kg) of cocaine (Bobzean et al., 2010). Consistent with the latter study, we found no difference in cocaine-induced CPP between male and female wild-type mice using either single or repeated conditioning.

While wild-type mice exhibited CPP following both single and repeated cocaine conditioning, we observed no significant CPP response in male or female mice lacking beta-endorphin following single cocaine conditioning, showing that the rewarding action of acute cocaine is abolished in the absence of beta-endorphin. We found that beta-endorphin is essential for the acquisition of CPP after repeated conditioning in male mice as well. Interestingly, female mice lacking beta-endorphin showed a robust CPP response after repeated conditioning with cocaine, indicating that endogenous beta-endorphin differentially contributes to the rewarding action of cocaine after its repeated administration. Although the underlying mechanism of this male/female difference regarding the role of beta-endorphin in cocaine reward after repeated cocaine conditioning is unknown at present, there may be some interactions between beta-endorphin and sex hormones to regulate the CPP response. Considering that the mesolimbic dopaminergic neurons have been implicated in the rewarding action of cocaine and that the activity of these neurons is regulated by sex hormones (Festa et al., 2004; Festa and Quinones-Jenab, 2004; Nazarian et al., 2004; Tobiansky et al., 2016) and beta-endorphin (Di Chiara and Imperato, 1988; Johnson and North, 1992), it is tempting to propose that neuronal inputs to the mesolimbic dopaminergic neurons containing beta-endorphin and sex hormones may have overlapping functions to facilitate neurotransmission along these neurons and one can compensate for the lack of the other. This explains why we observed a robust CPP response in female but not male mice lacking beta-endorphin since sex hormones, most likely estrogen, may have compensated for the lack of beta-endorphin in female wild-type mice. However, this convergent becomes functional only after repeated cocaine conditioning because the rewarding action of acute cocaine was blunted in both male and female mice lacking beta-endorphin. Thus, further studies are needed to delineate the underlying mechanism of the interaction between beta-endorphin and sex hormones in regulating cocaine reward following single versus repeated cocaine conditioning.

Drug craving and particularly relapse represents a serious issue in the treatment of drug addiction. Relapse can be easily triggered by any drug-associated cues, external environmental cues, or re-exposure to the drug itself (Bossert et al., 2013). A common reinstatement model in rodents uses either priming injections of the drug, exposure to different stressors, or contextual cues to test if the previously extinguished response can be reinstated (O’Brien et al., 1992). In the present study, we used a priming injection of cocaine (7.5 mg/kg) to determine if there was any difference in the reinstatement of cocaine-induced CPP between female and male mice and whether beta-endorphin would play a functional role in this response. We found that both male and female wild-type mice exhibited a significant CPP response following the priming dose of cocaine; yet, there was no difference in the magnitude of the CPP response between male and female mice on the reinstatement test day. Interestingly, however, cocaine failed to reinstate a CPP response in male or female mice lacking beta-endorphin. One may argue that male mice lacking beta-endorphin did not exhibit CPP, and thus one should not expect reinstatement of CPP in those mice. While this argument may be valid in some cases, the lack of reinstatement in knockout mice cannot be explained by the blunted CPP response in knockout mice because female mice lacking beta-endorphin expressed a robust CPP response after repeated conditioning with cocaine yet failed to exhibit a significant CPP response on the reinstatement test day. Thus, the present result provides the first evidence that endogenous beta-endorphin plays an essential role in processes leading to the reinstatement of cocaine CPP at least in C57BL/6 mice.

Research from Dr. Kreek’s laboratory has shown that animals exhibiting cocaine-induced CPP but not cocaine administration alone had elevated POMC mRNA levels in the hypothalamus (Zhou et al., 2012), raising the possibility that beta-endorphin may be needed for the acquisition and reinstatement of cocaine CPP. However, presently, it is unclear how beta-endorphin contributes to the processes leading to reinstatement. It is noteworthy to state that cocaine causes the release of beta-endorphin in the NAc (Amalric et al., 1987; Olive et al., 2001; Roth-Deri et al., 2003, 2004, 2006), a brain region where direct injection of beta-endorphin has been shown to induce CPP (Amalric et al., 1987). Furthermore, direct injection of beta-endorphin in the NAc elicited cocaine-seeking behaviors (Simmons and Self, 2009), raising the possibility that NAc may be the primary target where beta-endorphin exerts its regulatory actions on cocaine reward and reinstatement. Nevertheless, further research is needed to define how cocaine impacts the endogenous beta-endorphin and how endogenous beta-endorphin regulates the reinstatement of cocaine-induced CPP. Similarly, further research is needed to identify the brain region(s)/circuit(s) involved in these processes and define how endogenous beta-endorphin interacts with sex hormones to regulate cocaine-induced CPP and its reinstatement.

In summary, our results indicate that beta-endorphin is involved in the rewarding actions of acute cocaine in both male and female mice. However, there was a sex-related difference in this response in mice lacking beta-endorphin, in which female but not male mice lacking beta-endorphin exhibited CPP following repeated conditioning with cocaine. Nonetheless, re-exposure to cocaine failed to reinstate the CPP response in mice lacking beta-endorphin regardless of the sex of mice. Collectively, the present results indicate that there is an interaction between endogenous opioid peptide beta-endorphin and sex of mice in the acquisition of CPP after repeated conditioning with cocaine but not reinstatement of cocaine CPP.

## Data Availability Statement

The raw data supporting the conclusions of this article will be made available by the authors, without undue reservation.

## Ethics Statement

The animal study was reviewed and approved by the Animal Care and Use Committee at Western University of Health Sciences (Pomona, CA, United States).

## Author Contributions

PS conducted the experiments and prepared the first draft of the manuscript. KL designed the project, analyzed the data, and finalized the manuscript. Both authors contributed to the article and approved the submitted version.

## Conflict of Interest

The authors declare that the research was conducted in the absence of any commercial or financial relationships that could be construed as a potential conflict of interest.

## Publisher’s Note

All claims expressed in this article are solely those of the authors and do not necessarily represent those of their affiliated organizations, or those of the publisher, the editors and the reviewers. Any product that may be evaluated in this article, or claim that may be made by its manufacturer, is not guaranteed or endorsed by the publisher.
