# Supplementary material for: The Role of Beta-Endorphin in Cocaine-Induced Conditioned Place Preference, Its Extinction, and Reinstatement in Male and Female Mice
Source: Front Behav Neurosci. 2021 Dec 10;15:763336. doi: 10.3389/fnbeh.2021.763336 (PMC8702804; doi:10.3389/fnbeh.2021.763336)

Supplemental Figures (Dot Blot presentations of the Data)

Fig. 1S

CPP was blunted in male mice lacking beta-endorphin compared to their wild-type controls following single and repeated conditioning with cocaine (15 mg/kg) as well as on the reinstatement test day following a challenge dose of cocaine (7.5 mg/kg, i.p.). Data are mean (± SEM) of the amount of time that animals (n = 6-8 mice per genotype) spent in the drug-paired chamber (DPCh) versus vehicle-paired chamber (VPCh) before (day 1; D1) and after single (day 3; D3) and repeated (day 5; D5) conditioning with cocaine as well as on the extinction and reinstatement test days. ; *P<0.05; **P<0.01; ***P<0.001 vs. its respective VPCh; +P<0.05 vs. mice lacking beta-END on that test day

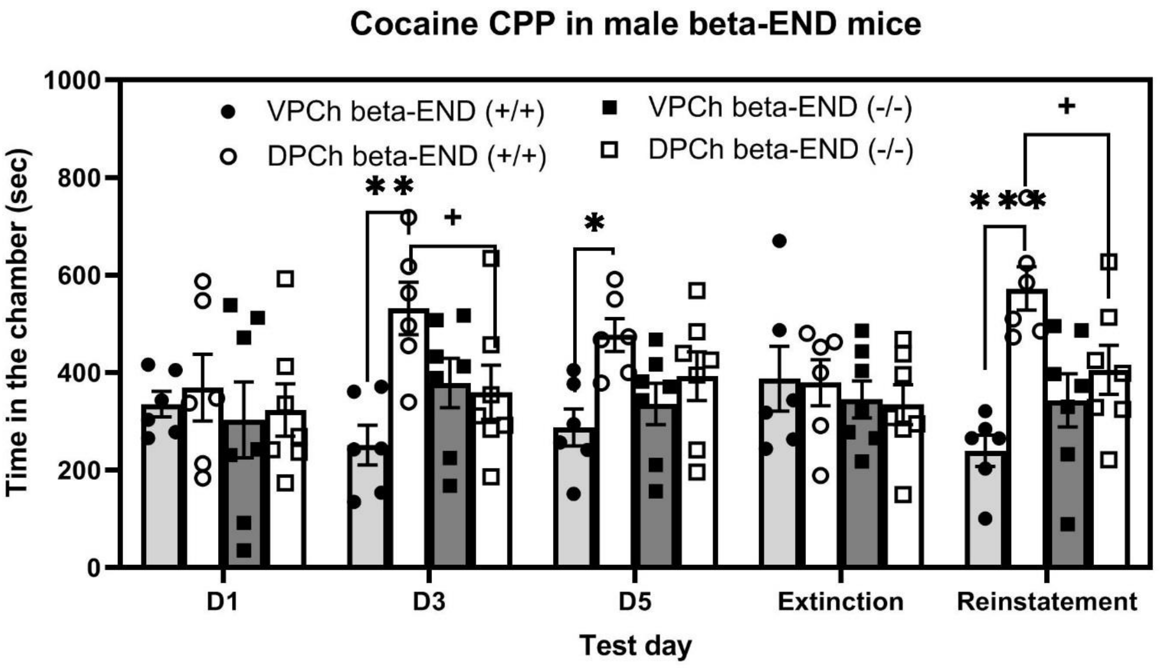

Fig. 2S

The CPP response was blunted in female mice lacking beta-endorphin compared to their wild-type controls following single but not repeated conditioning with cocaine (15 mg/kg) as well as on the reinstatement test day. Data are mean (± SEM) of the amount of time that mice (n = 8 mice per genotype) spent in the drug-paired chamber (DPCh) versus vehicle-paired chamber (VPCh) on day 1 (D1) and after single (day 3; D3) and repeated (day 5, D5) conditioning with cocaine as well as on the extinction and reinstatement test days; *P<0.05; **P<0.01 vs. their respective VPCh on that day.

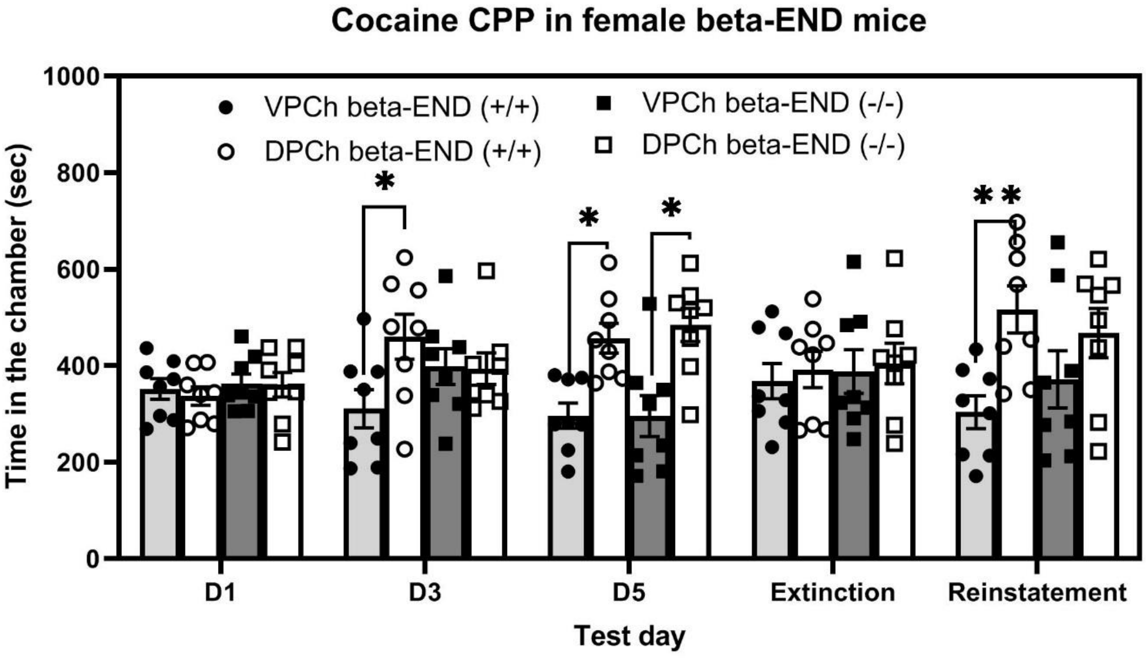

Supplement: Supplementary file 1 [file Data_Sheet_1.pdf]
